# A nosocomial cluster of vancomycin resistant enterococci among COVID-19 patients in an intensive care unit

**DOI:** 10.1186/s13756-020-00820-8

**Published:** 2020-09-22

**Authors:** Stefanie Kampmeier, Hauke Tönnies, Carlos L. Correa-Martinez, Alexander Mellmann, Vera Schwierzeck

**Affiliations:** Institute of Hygiene, University Hospital Münster, Robert-Koch-Straße 41, 48149 Münster, Germany

**Keywords:** *vanB*, Nosocomial VRE cluster, Healthcare-associated infection, Surface contamination, Intensive care unit, COVID-19

## Abstract

**Background:**

Currently, hospitals have been forced to divert substantial resources to cope with the ongoing coronavirus disease 2019 (COVID-19) pandemic. It is unclear if this situation will affect long-standing infection prevention practices and impact on healthcare associated infections. Here, we report a nosocomial cluster of vancomycin-resistant enterococci (VRE) that occurred on a COVID-19 dedicated intensive care unit (ICU) despite intensified contact precautions during the current pandemic. Whole genome sequence-based typing (WGS) was used to investigate genetic relatedness of VRE isolates collected from COVID-19 and non-COVID-19 patients during the outbreak and to compare them to environmental VRE samples.

**Methods:**

Five VRE isolated from patients (three clinical and two screening samples) as well as 11 VRE and six vancomycin susceptible *Enterococcus faecium* (*E. faecium*) samples from environmental sites underwent WGS during the outbreak investigation. Isolate relatedness was determined using core genome multilocus sequence typing (cgMLST).

**Results:**

WGS revealed two genotypic distinct VRE clusters with genetically closely related patient and environmental isolates. The cluster was terminated by enhanced infection control bundle strategies.

**Conclusions:**

Our results illustrate the importance of continued adherence to infection prevention and control measures during the COVID-19 pandemic to prevent VRE transmission and healthcare associated infections.

## Introduction

In December 2019, severe acute respiratory syndrome coronavirus 2 (SARS-CoV-2) was discovered in Wuhan City, China and rapidly developed into a pandemic that poses a serious threat to health care systems worldwide [1–3]. It soon became apparent that even in developed countries COVID-19 will set an unprecedented challenge to hospitals due to a high influx of critically ill patients, demand for mechanical ventilation beds and shortage of personal protective equipment (PPE) [4–6]. Understandably, major staff and financial resources have been diverted to COVID-19 outbreak management in hospitals, especially in regions with high case numbers [7]. One concern among the infection prevention community is that the COVID-19 pandemic will cause “collateral damage” to long-established infection control measures including the prevention of healthcare-associated infections [8]. So far, it remains unknown if the present diversion of hospital resources and changes in infection control practices will translate into an increase of nosocomial transmissions, in particular of multidrug resistant organisms (MDRO), during the COVID-19 pandemic or if the increased efforts to improve infection control measures will have a positive effect on nosocomial MDRO transmissions [9, 10].

In recent years, the incidence of VRE infections has increased worldwide, including in Germany [11]. VRE infections are associated with significant mortality, in particular in patients with multiple co-morbidities and ICU patients [12, 13]. Nosocomial transmission of VRE is frequent, partly because enterococci are resilient and survive for prolonged periods on inanimate surfaces i.e. medical equipment, work top surfaces, furniture [14, 15]. Here, we describe a nosocomial cluster of VRE that involved three COVID-19 patients as well as two SARS-CoV-2 negative ICU patients.

## Methods

### Clinical setting and infection control measures

The University Hospital Münster (UKM) is a 1800-bed care centre admitting ca. Seventy-four thousand patients per year and treating up to 140 patients on 11 ICUs or intermediate care units. During the COVID-19 pandemic, one of the ICUs was separated into two subunits, treating COVID-19 patients and non-COVID-19 patients, respectively. Enhanced infection control measures were put in place for confirmed and suspected COVID-19 patients, including isolation in the COVID-19 ward and the use of PPE (gloves, gowns, FFP2 masks and safety googles). In April 2020, VRE from clinically relevant samples (blood culture samples and pleural drainage) were detected in three patients from both ICU subunits. As this rate exceeded the baseline of three VRE infections per year on this ward, an outbreak investigation was initiated and – in accordance to the German guideline published in 2018 - a bundle strategy for terminating VRE transmission was implemented [16]. This bundle comprised a point prevalence screening among all patients, a VRE screening on admission and once a week for every patient, environmental sampling to detect VRE contamination of patient surroundings, contact precautions including the use of PPE (gloves and gowns) and patient isolation. Isolation of patients could be discontinued if three anorectal swab samples collected in at least a one-week interval without administering antibiotic therapy during this time were negative for VRE. In addition, intensified surface disinfection was established using an alkylamine, Incidin™ plus 0.5% (ECOLAB Healthcare, Monheim am Rhein, Germany), focussing in particular on patient rooms, nurses’ rooming homes, storage rooms and staff rest rooms. Moreover, hand hygiene training was performed among nurses, physicians and cleaning personnel.

### Environmental sampling and testing method

Environmental sampling was performed using sterile packaged polywipes (mwe, Corsham, Wiltshire, UK) on contact surfaces and incubating them in Tryptic Soy Broth + lecithin tween (LT) (Merck Millipore, Eppelheim, Germany) for 24 h at 37 °C. Ten μL of this broth were streaked onto blood agar and VRE selective agar and incubated for 24 h at 37 °C. Suspected colonies were subcultured on blood agar and species identification was performed by MALDI-TOF-MS (Bruker Corporation, Bremen, Germany). Susceptibility testing for vancomycin was performed using Etest® (Bestbion GmbH, Liofilchem, Italy). Susceptibility testing for methicillin was performed using Cefoxitin antimicrobial susceptibility disks (Oxoid, ThermoFisher Scientific,Waltham, USA). Results wereevaluated in accordance with the EUCAST standards for clinical breakpoints (version 10.0).

### Whole genome sequence-based typing

Confirmed *E. faecium* isolates were subjected to WGS using the Illumina MiSeq platform (Illumina Inc., San Diego, USA). Specifically, DNA was extracted using a glass-bead-based method [17] and 1 ng DNA was introduced into library preparation with a Nextera XT DNA sample preparation kit (Illumina) and paired-end sequenced with a MiSeq Reagent kit v2 250 bp (Illumina) with an average insertion size of 300 bp. Libraries were scaled to reach 100-fold sequencing coverage for an average genome size of 3 MB. Only sequencing runs that fulfilled the manufacturers’ specifications (Illumina) with respect to cluster density and Q30 were further analyzed. After sequencing, the raw reads were quality-trimming and de novo assembled using the SKESA algorithm with default parameters [18] that is implemented in the Ridom SeqSphere^+^ software version 7.0.1 (Ridom GmbH, Münster, Germany) [19]. For subsequent core genome multilocus sequence typing (cgMLST), the previously published cgMLST scheme for *E. faecium* was used [20]. Here, every sample had to have ≥95% extracted cgMLST targets, otherwise sequencing had to be repeated. Based on the allelic profiles from cgMLST; we constructed a minimum spanning tree to display the genetic relationships between isolates, a minimum spanning tree algorithm was applied. We rated isolates differing ≤3 alleles as closely related with a high likelihood of resulting from a clonal transmission event [21, 22]. In addition, *van*-genotypes and MLST sequence types (ST) were extracted from the WGS data in silico using SeqSphere^+^.

## Results

### Outbreak management and environmental sampling

**Fig. 1:**
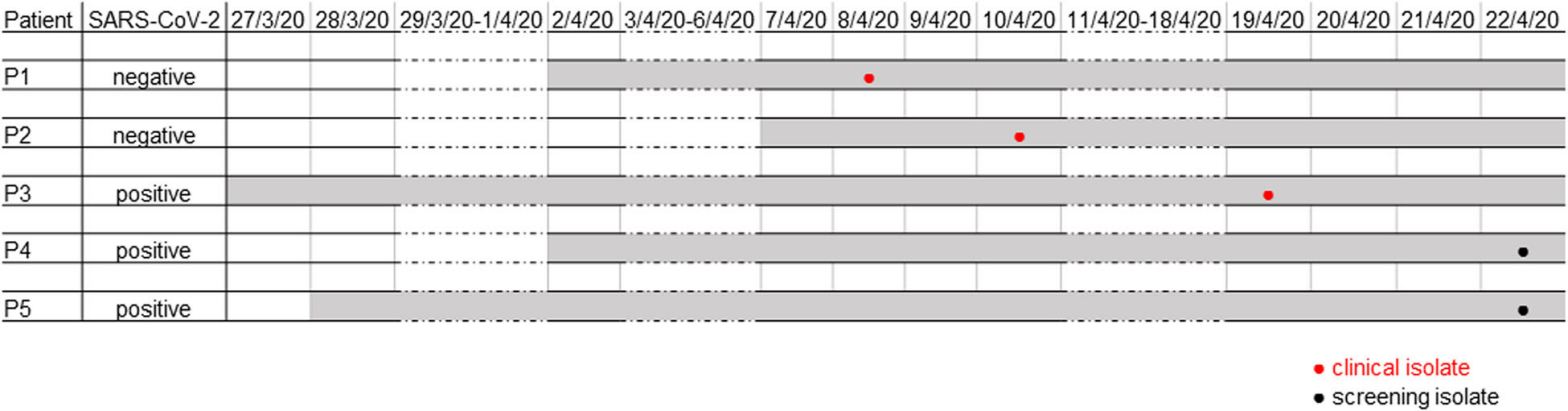
Timeline of the nosocomial VRE cluster. VRE positive patients are illustrated chronologically. Each line represents the timeline of a single patient. Grey shading of lines indicates duration of residence on ICU. VRE isolates derived from clinical (*red*) or screening samples (*black*) are shown as dots within the lines

After VRE has been detected in clinical samples from three patients from both ICU subunits in April 2020, patient screening for VRE was initiated as part of an outbreak investigation. Patient screening revealed two additional VRE positive patients (Fig. 1). Environmental sampling on relevant hand contact surfaces (e.g. nursing workspace, PC, monitor, infusion stand etc., *n* = 30) resulted in 17 swab sampling probes that were positive for *E. faecium*, of which 11 were VRE. Moreover, in 18 samples other nosocomial pathogens such as *S. aureus* (methicillin susceptible) (*n* = 6), *E. faecalis* (*n* = 6), *Enterobacterales* (*n* = 3) and nonfermenters (*n* = 3) were identified.

After establishing the previously mentioned infection control bundle strategy the cluster could be terminated immediately. No further VRE infections or nosocomial colonisations with this clone occurred to date, 5 months after the outbreak.

### Whole genome sequence-based typing

**Fig. 2:**
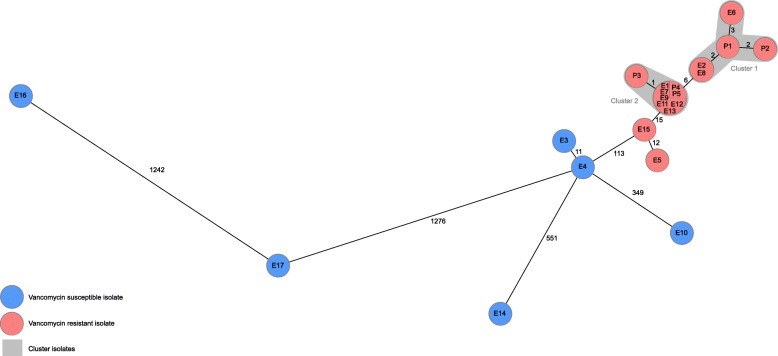
Minimum spanning tree of detected *E. faecium*. Minimum spanning tree of 17 environmental (E) and five patient (P) vancomycin resistant (*red*) and vancomycin susceptible (*blue)* isolates illustrating their genotypic relationship based on up to 1423 cgMLST target genes, pairwise ignore missing values. Every circle represents one genotype, the size of circles correlates with the number of identical genotypes. Grey colouring indicates close genetic relation (≤ 3 alleles differing between two genotypes)

All five patient samples, 11 environmental VRE and – for comparison – also the six susceptible environmental *E. faecium* isolates were subjected to WGS. The mean sequencing coverage was 133x (53-200x), the number of contigs ranged from 140 to 483 (mean = 243), the mean assembly size was 2.955,382 bp (range, 2.630,395–3.019,336 bp), and on average 98.6% (95.2–99.2%) of the 1423 cgMLST targets were detected. The analysis resulted in two clusters of five and nine closely related strains (≤ 3 alleles differing between genotypes) and eight singletons. Cluster 1 (C1) and cluster 2 (C2) are distantly related to each other (6 alleles differing between most similar genotypes). Both clusters C1 (P1, P2, E2, E6, E8) and C2 (P3, P4, P5, E1, E7, E9, E11, E12, E13) comprise patient and environmental isolates (Fig. 2). Interestingly, the VRE samples of the two SARS-CoV-2 negative patients are part of C1 while all samples collected from COVID-19 patients were C2. All samples of C1 and C2 were identified as ST117 and *vanB*. In all 16 VRE detected during the outbreak *vanB* could be detected. Most prevalent MLST ST was ST117 in 18 (81.8%) strains, of which 16 were tested vancomycin resistant and two vancomycin susceptible. Additional information of distribution of *van*-genes and MLST ST in patient and environmental isolates is displayed in Table 1.

**Table 1:** *E. faecium* isolates derived from patients (P) and environmental sites (E)

Isolate no.	MLST ST	***Van-***genotype	Origin of sample or sampling location
P1	ST117	*van*B	blood culture
P2	ST117	*van*B	pleural drainage fluid
P3	ST117	*van*B	blood culture
P4	ST117	*van*B	screening sample
P5	ST117	*van*B	screening sample
E1	ST117	*van*B	non-isolation ward, room 9
E2	ST117	*van*B	non-isolation ward, room 9 (anteroom)
E3	ST117	–	non-isolation ward, hallway (furniture)
E4	ST117	–	non-isolation ward, clean utility room (designated *iv* preparation station)
E5	ST117	*van*B	holding area (furniture)
E6	ST117	*van*B	non-isolation ward, office space (work surfaces)
E7	ST117	*van*B	COVID-19 isolation ward, window
E8	ST117	*van*B	COVID-19 isolation ward, PC
E9	ST117	*van*B	COVID-19 isolation ward, intermediate life support ward emergency bag
E10	ST80	–	COVID-19 isolation ward, intermediate life support ward emergency bag
E11	ST117	*van*B	COVID-19 isolation ward, anteroom
E12	ST117	*van*B	COVID-19 isolation ward, anteroom (clean)
E13	ST117	*van*B	COVID-19 isolation ward, clean utility room
E14	ST324	–	COVID-19 isolation ward, clean utility room
E15	ST117	*van*B	COVID-19 isolation ward, nurses’ station (furniture)
E16	ST107	–	COVID-19 isolation ward, nurses’ station (PC)
E17	ST361	–	COVID-19 isolation ward, office space (work surfaces)

## Discussion

In the present study, we demonstrated the transmission of VRE in an ICU by WGS during the ongoing COVID-19 pandemic. The close genetic relatedness between environmental VRE samples and patient VRE isolates indicated a role for contaminated surfaces in this cluster. Indirect contact has been frequently described as route of transmission for VRE in the hospital setting, due to the persistence of the bacterium [14, 22]. WGS in combination with epidemiological data analysis suggests that contaminated surfaces were also involved in ongoing transmission during this reported cluster. Analysis of the WGS data showed that the VRE isolates of patients in the COVID-19 isolation ward differed by 6 alleles to the isolates of SARS-CoV-2 negative patients. However, based on epidemiological data, it is difficult to draw conclusion if this represents two separate transmission events. The majority of clones identified during the cluster were MLST ST117 and *vanB* positive. These clones are known to cause nosocomial outbreaks and match the current VRE epidemiology in German hospitals [23]. Enhanced infection control bundles were effective in terminating the transmission chains.

Intriguingly, this cluster started despite intensified contact precautions, including PPE and isolation of COVID-19 confirmed or suspected patients. Environmental sampling uncovered not only VRE, but also a variety of other nosocomial pathogens such as vancomycin susceptible enterococci, *S. aureus*, nonfermenters and *Enterobacterales.* This illustrates the importance of hand and surface disinfection within infection prevention bundles. At the time of the cluster, minimum staffing requirements in German hospitals have been suspended in order to cope with the ongoing COVID-19 pandemic. This situation is worrying as reduced personal is associated with poor adherence to infection control measures [24, 25]. Surveillance strategies will be important to elucidate any increase in hospital acquired infections and transmission events during this challenging situation. Infection control specialists need to raise awareness for long-established infection control routine measures as part of COVID-19 management in order to reduce mortality and morbidity associated with nosocomial infections.

## Conclusions

Our observation constitutes initial evidence of a feared yet unassessed negative impact of resource diversion on the epidemiology of MDRO, highlighting the need for continued infection prevention and control measures during the time of the COVID-19 pandemic to prevent transmissions and healthcare associated infections with MDRO.

## Data Availability

Genome assemblies of the analysed strains are available under NCBI BioProject ID PRJNA662979.

## Abbreviations

COVID-19: Coronavirus disease 2019
*E. faecium*: *Enterococcus faecium*
ICU: Intensive care unit
MDRO: Multi-drug resistant organisms
PPE: Personal protective equipment
SARS-CoV-2: Severe acute respiratory syndrome coronavirus 2
VRE: Vancomycin-resistant enterococci
WGS: Whole genome sequencing

## References

[1] Chan JF-W, Yuan S, Kok K-H, To KK-W, Chu H, Yang J (2020). A familial cluster of pneumonia associated with the 2019 novel coronavirus indicating person-to-person transmission: a study of a family cluster. Lancet.

[2] Zhou P, Yang X-L, Wang X-G, Hu B, Zhang L, Zhang W (2020). A pneumonia outbreak associated with a new coronavirus of probable bat origin. Nature.

[3] Verelst F, Kuylen E, Beutels P (2020). Indications for healthcare surge capacity in European countries facing an exponential increase in coronavirus disease (COVID-19) cases, March 2020. Euro Surveill.

[4] Remuzzi A, Remuzzi G (2020). COVID-19 and Italy: what next?. Lancet.

[5] White DB, Lo B (2020). A framework for rationing ventilators and critical care beds during the COVID-19 pandemic. JAMA.

[6] Alhazzani W, Møller MH, Arabi YM, Loeb M, Gong MN, Fan E (2020). Surviving Sepsis campaign: guidelines on the management of critically ill adults with coronavirus disease 2019 (COVID-19). Intensive Care Med.

[7] Carenzo L, Costantini E, Greco M, Barra FL, Rendiniello V, Mainetti M (2020). Hospital surge capacity in a tertiary emergency referral Centre during the COVID-19 outbreak in Italy. Anaesthesia.

[8] Stevens MP, Doll M, Pryor R, Godbout E, Cooper K, Bearman G. Impact of COVID-19 on traditional healthcare-associated infection prevention efforts. Infect Control Hosp Epidemiol. 2020:1–2. 10.1017/ice.2020.141. 10.1017/ice.2020.141 PMC7188960 32297849

[9] Rawson TM, Moore LSP, Castro-Sanchez E, Charani E, Davies F, Satta G (2020). COVID-19 and the potential long-term impact on antimicrobial resistance. J Antimicrob Chemother.

[10] Vaughn VM, Gandhi T, Petty LA, Patel PK, Prescott HC, Malani AN, et al. Empiric antibacterial therapy and community-onset bacterial co-infection in patients hospitalized with COVID-19: a multi-hospital cohort study. Clin Infect Dis. 2020. 10.1093/cid/ciaa1239. 10.1093/cid/ciaa1239 PMC7499526 32820807

[11] Pfaller MA, Cormican M, Flamm RK, Mendes RE, Jones RN (2019). Temporal and geographic variation in antimicrobial susceptibility and resistance patterns of enterococci: results from the SENTRY antimicrobial surveillance program, 1997-2016. Open Forum Infect Dis.

[12] Prematunge C, MacDougall C, Johnstone J, Adomako K, Lam F, Robertson J (2016). VRE and VSE bacteremia outcomes in the era of effective VRE therapy: a systematic review and meta-analysis. Infect Control Hosp Epidemiol.

[13] Kim YJ, Kim SI, Kim YR, Lee JY, Park YJ, Kang MW (2012). Risk factors for vancomycin-resistant enterococci infection and mortality in colonized patients on intensive care unit admission. Am J Infect Control.

[14] Wagenvoort JHT, de Brauwer EIGB, Penders RJR, Willems RJ, Top J, Bonten MJ (2011). Environmental survival of vancomycin-resistant enterococcus faecium. J Hosp Infect.

[15] Erb S, Frei R, Dangel M, Widmer AF (2017). Multidrug-resistant organisms detected more than 48 hours after hospital admission are not necessarily hospital-acquired. Infect Control Hosp Epidemiol.

[16] Empfehlung der Kommission für Krankenhaushygiene und Infektionsprävention (KRINKO) beim Robert Koch-Institut. Hygienemaßnahmen zur Prävention der Infektion durch Enterokokken mit speziellen Antibiotikaresistenzen. Available from: URL: https://www.rki.de/DE/Content/Infekt/Krankenhaushygiene/Kommission/Downloads/Enterokokken_Rili.pdf?__blob=publicationFile. Accessed 10 Sept 2020. 10.1007/s00103-018-2811-2 30229318

[17] Köser CU, Fraser LJ, Ioannou A, Becq J, Ellington MJ, Holden MTG (2014). Rapid single-colony whole-genome sequencing of bacterial pathogens. J Antimicrob Chemother.

[18] Souvorov A, Agarwala R, Lipman DJ (2018). SKESA: strategic k-mer extension for scrupulous assemblies. Genome Biol.

[19] Mellmann A, Bletz S, Böking T, Kipp F, Becker K, Schultes A (2016). Real-time genome sequencing of resistant Bacteria provides precision infection control in an institutional setting. J Clin Microbiol.

[20] de Been M, Pinholt M, Top J, Bletz S, Mellmann A, van Schaik W (2015). Core genome multilocus sequence typing scheme for high- resolution typing of enterococcus faecium. J Clin Microbiol.

[21] Correa-Martinez CL, Stollenwerk VB, Kossow A, Schaumburg F, Mellmann A, Kampmeier S (2019). Risk factors for long-term vancomycin-resistant enterococci persistence-a prospective longitudinal study. Microorganisms.

[22] Correa-Martinez CL, Tönnies H, Froböse NJ, Mellmann A, Kampmeier S (2020). Transmission of Vancomycin-resistant enterococci in the hospital setting: uncovering the patient-environment interplay. Microorganisms.

[23] Liese J, Schüle L, Oberhettinger P, Tschörner L, Nguyen T, Dörfel D (2019). Expansion of Vancomycin-Resistant *Enterococcus faecium* in an Academic Tertiary Hospital in Southwest Germany: a large-scale whole-genome-based outbreak investigation. Antimicrob Agents Chemother.

[24] Hugonnet S, Chevrolet J-C, Pittet D (2007). The effect of workload on infection risk in critically ill patients. Crit Care Med.

[25] Bowblis JR (2011). Staffing ratios and quality: an analysis of minimum direct care staffing requirements for nursing homes. Health Serv Res.

